# Supplementary material for: Roughness Evolution Induced by Third-Body Wear
Source: Tribol Lett. 2024 Mar 8;72(2):37. doi: 10.1007/s11249-024-01833-9 (PMC10924009; doi:10.1007/s11249-024-01833-9)
Supplement: Supplementary file 1 — (pdf 6804 KB) [file 11249_2024_1833_MOESM1_ESM.pdf]

Supplementary material

A Extra surface evolution plot

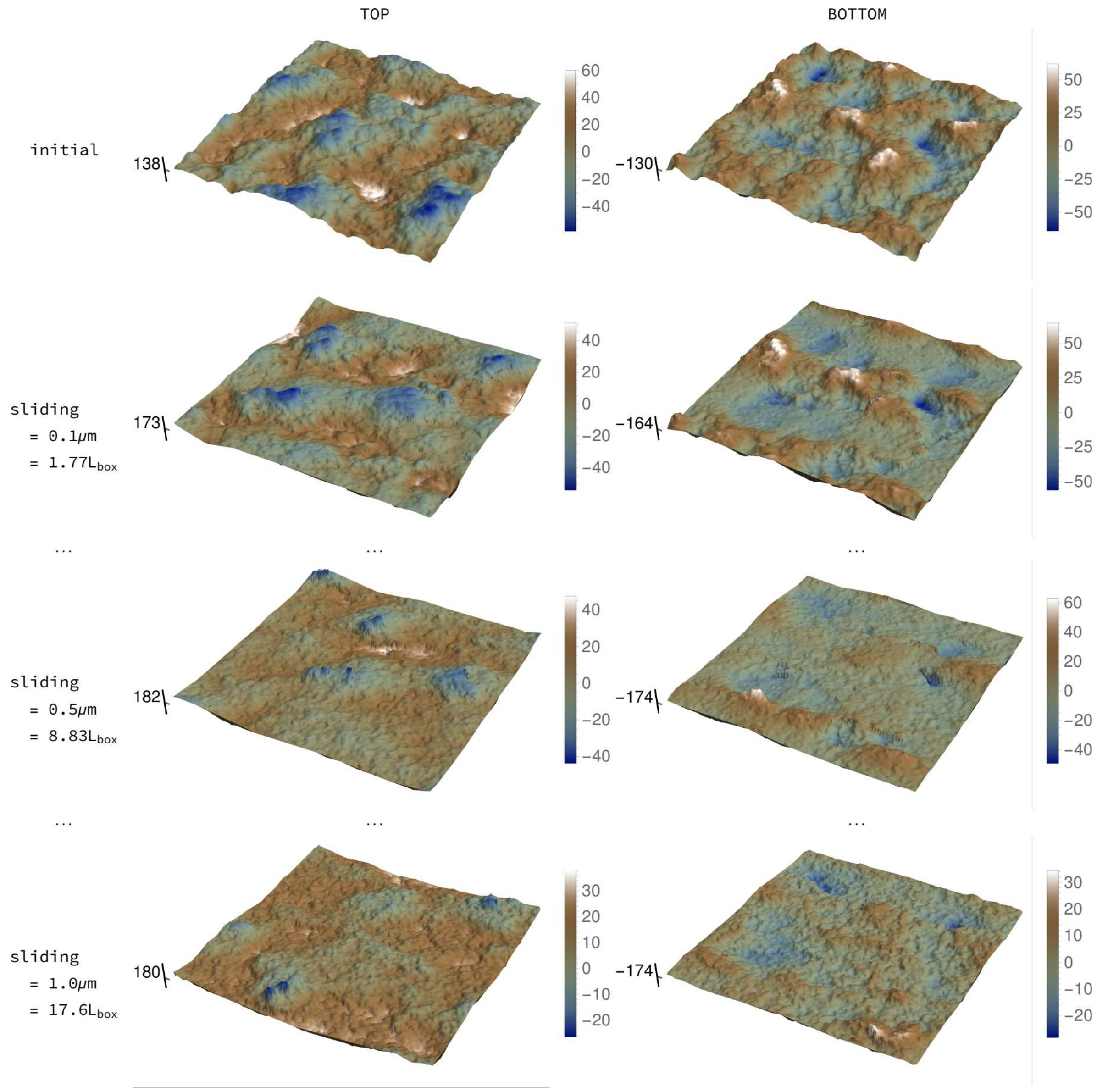

Figure A.1: Snapshots of surface evolution (units in Å unless otherwise stated): silicon surfaces (top and bottom) initially rough, single-crystalline bulk. Corresponding Hurst exponent evolution in Figure 4c. Note changing scales. Vertical axes mark the (evolving) mean height of each surface. In either surfaces, white means topographical features “bulging out”, while blue means penetrating into the surface bulk.

B 1D line scans: computing final Hurst

B.1 Flat surface with nanocrystalline bulk

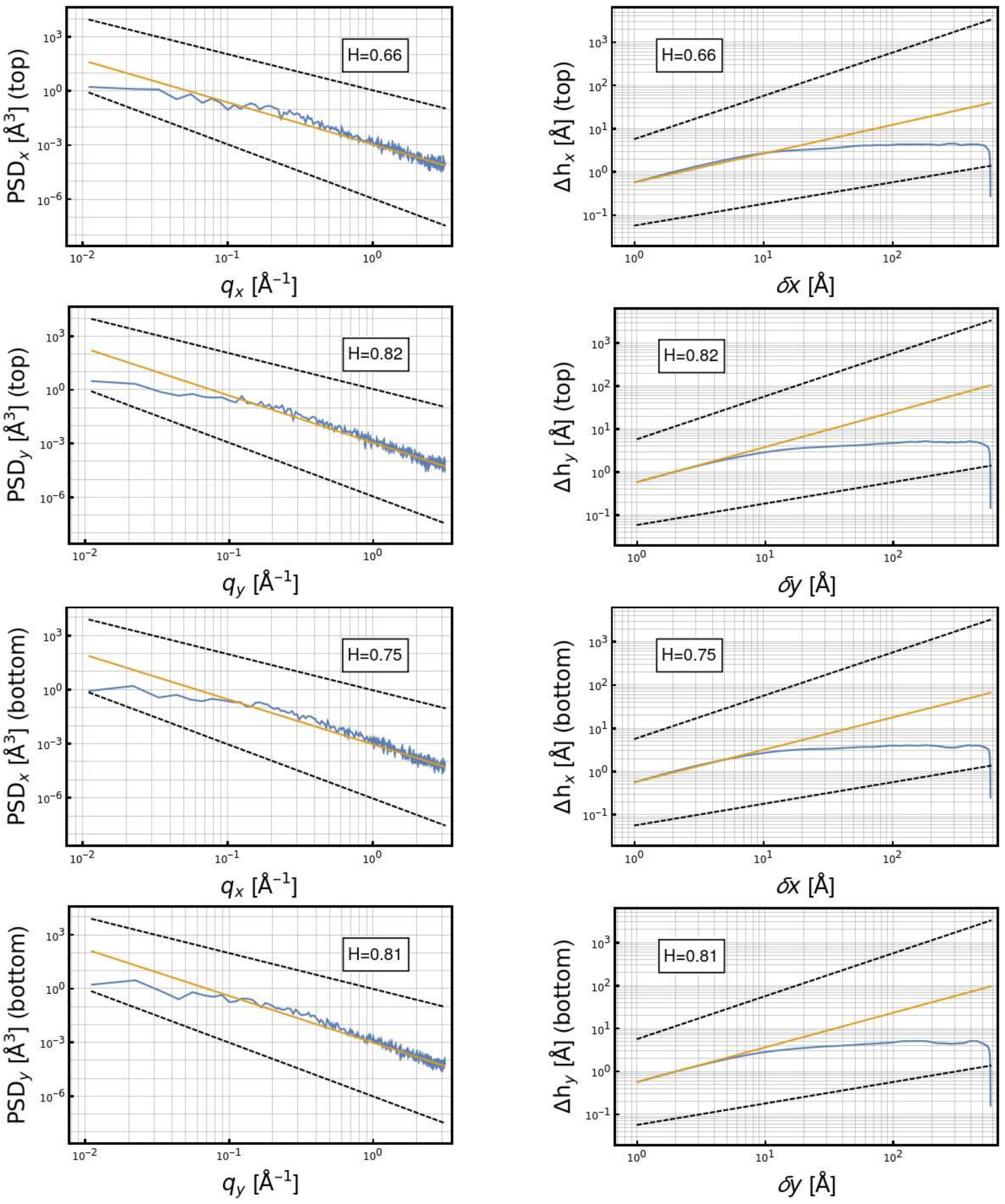

Figure B.1: 1D scans by the end of sliding, flat surface with nanocrystalline bulk. The left column shows the PSDs, the right column the heigh–height correlation.

B.2 Rough surface with nanocrystalline bulk

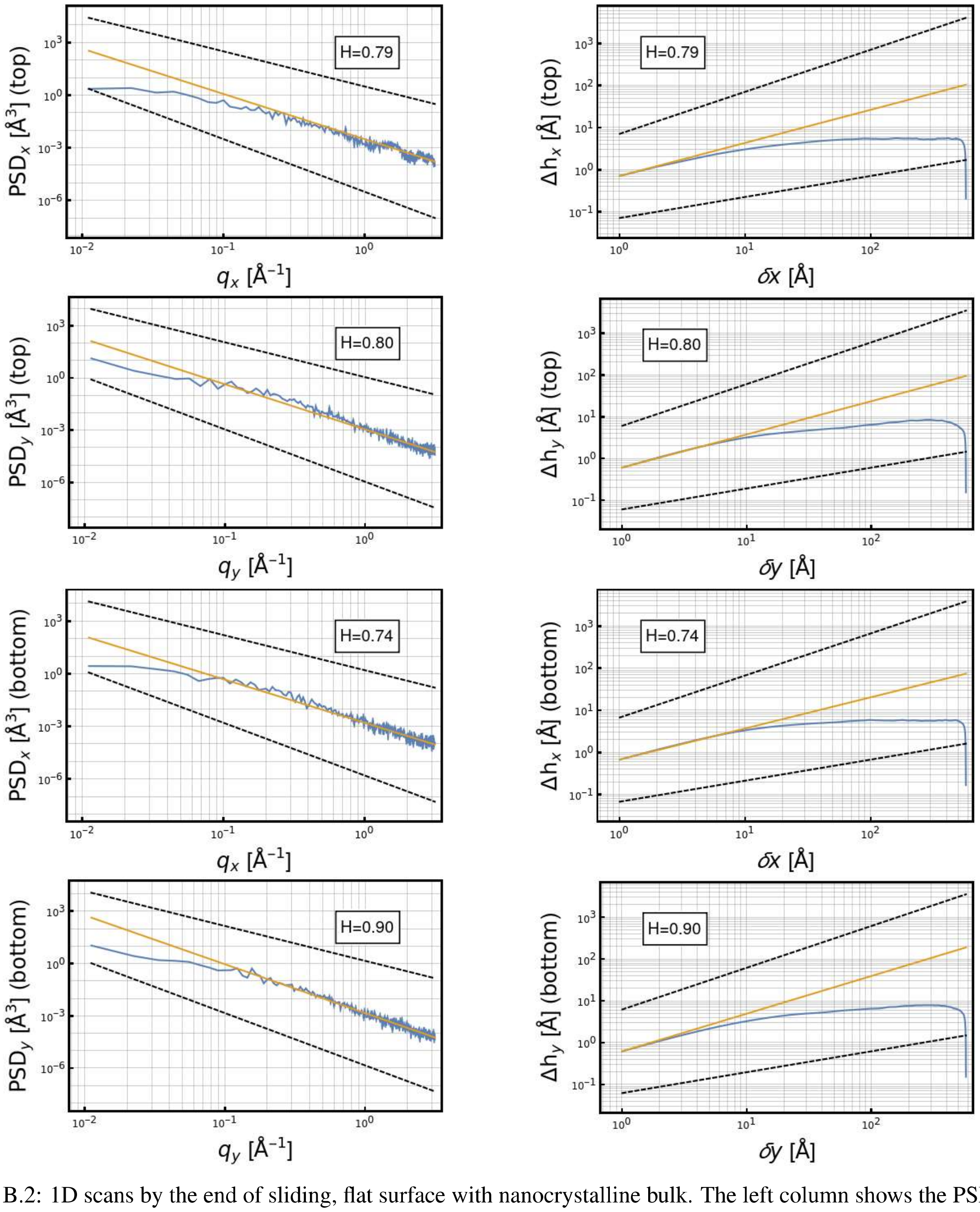

Figure B.2: 1D scans by the end of sliding, flat surface with nanocrystalline bulk. The left column shows the PSDs, the right column the heigh–height correlation.

B.3 Rough surface with monocrystalline bulk

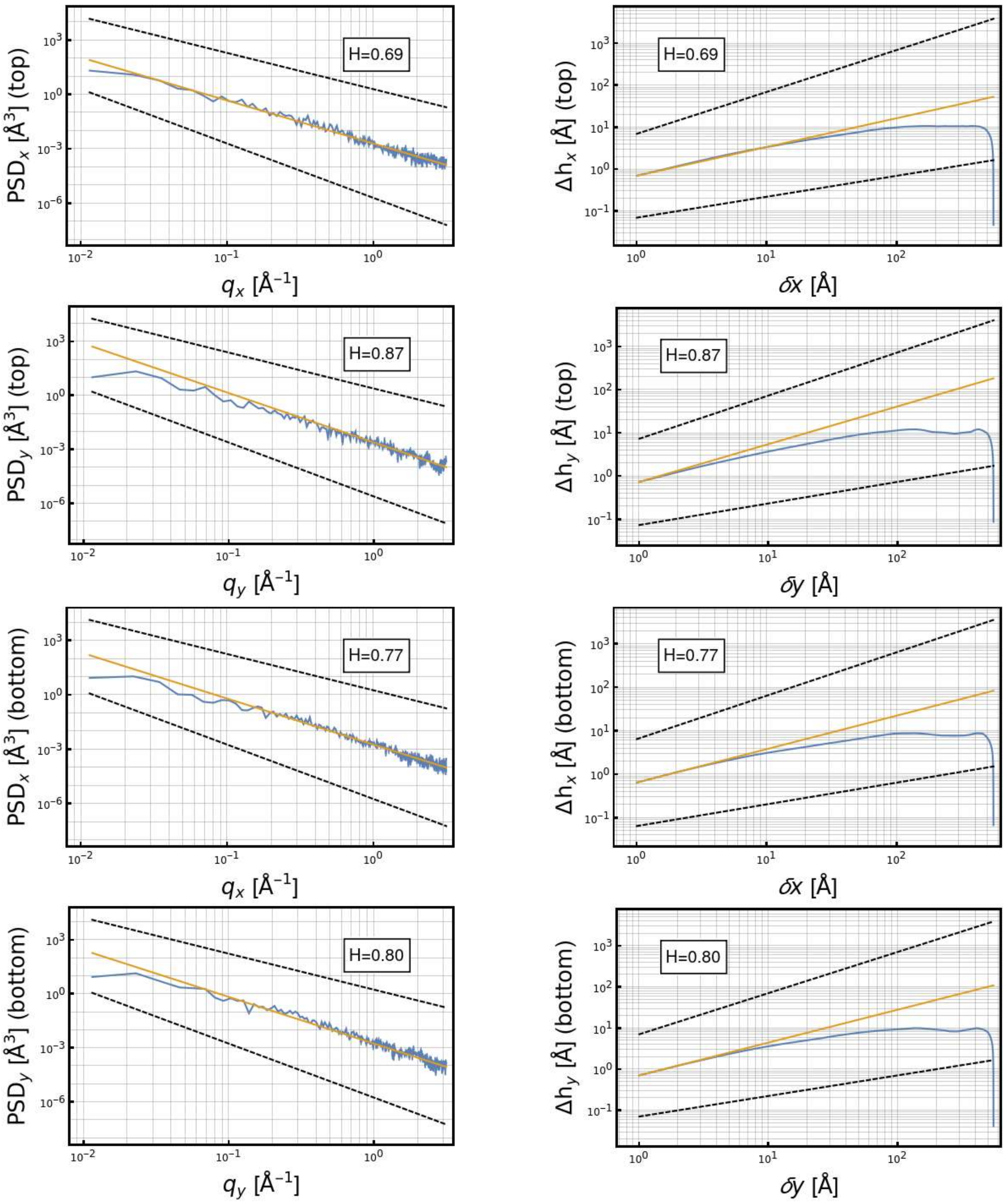

Figure B.3: 1D scans by the end of sliding, flat surface with monocrystalline bulk. The left column shows the PSDs, the right column the height–height correlation.

C Power spectral density plots of bottom surfaces (top ones are similar)

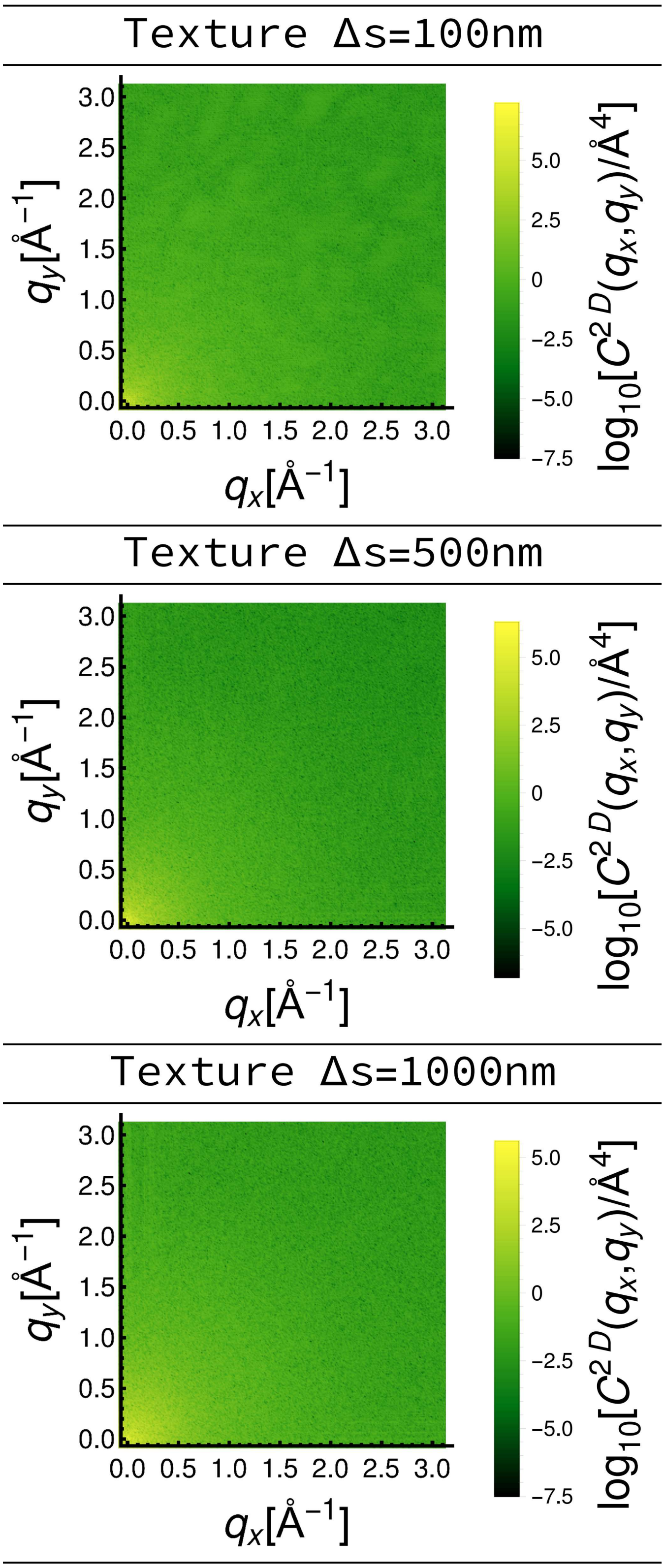

Figure C.1: Quadrant $q_x, q_x > 0$ of PSD initially-flat Si bottom surfaces (nanocrystalline bulk) to assess texture evolution.

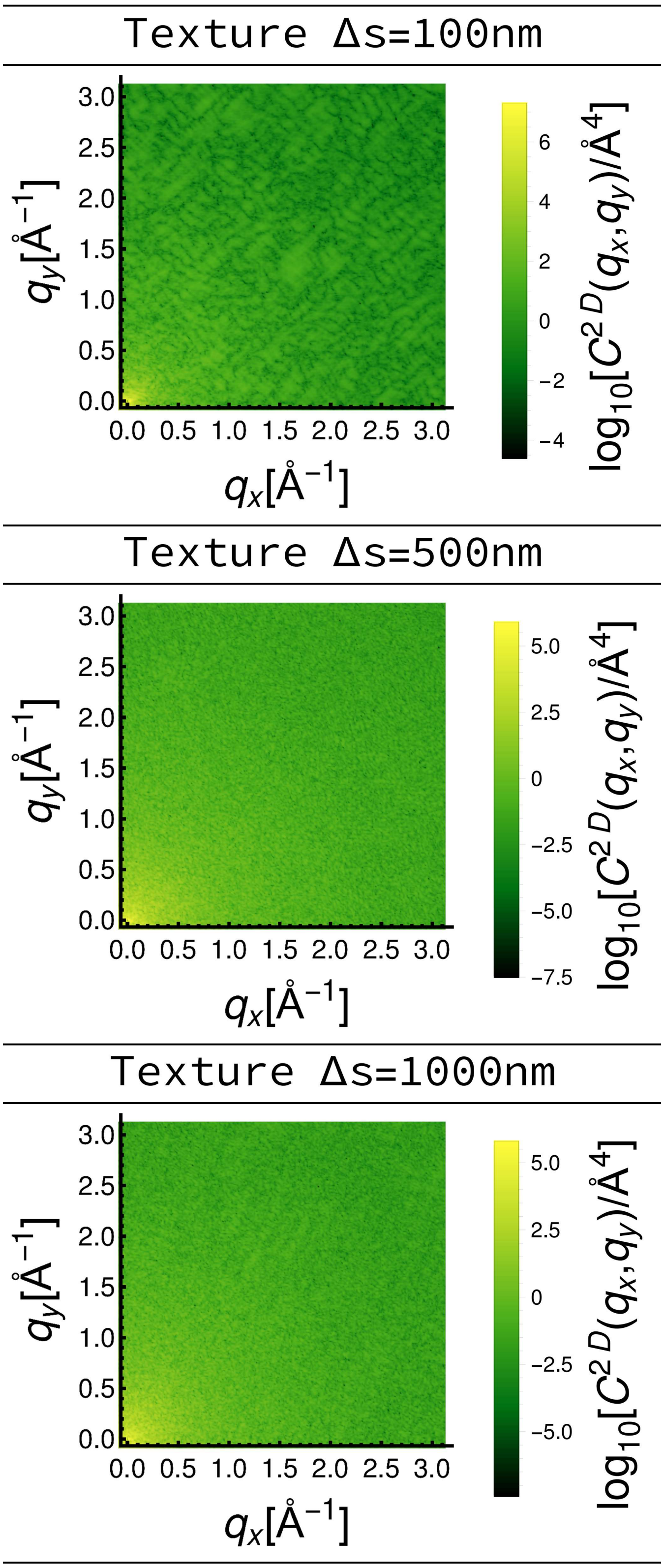

Figure C.2: Quadrant $q_x, q_x > 0$ of PSD initially-rough Si bottom surfaces (nanocrystalline bulk) to assess texture evolution.

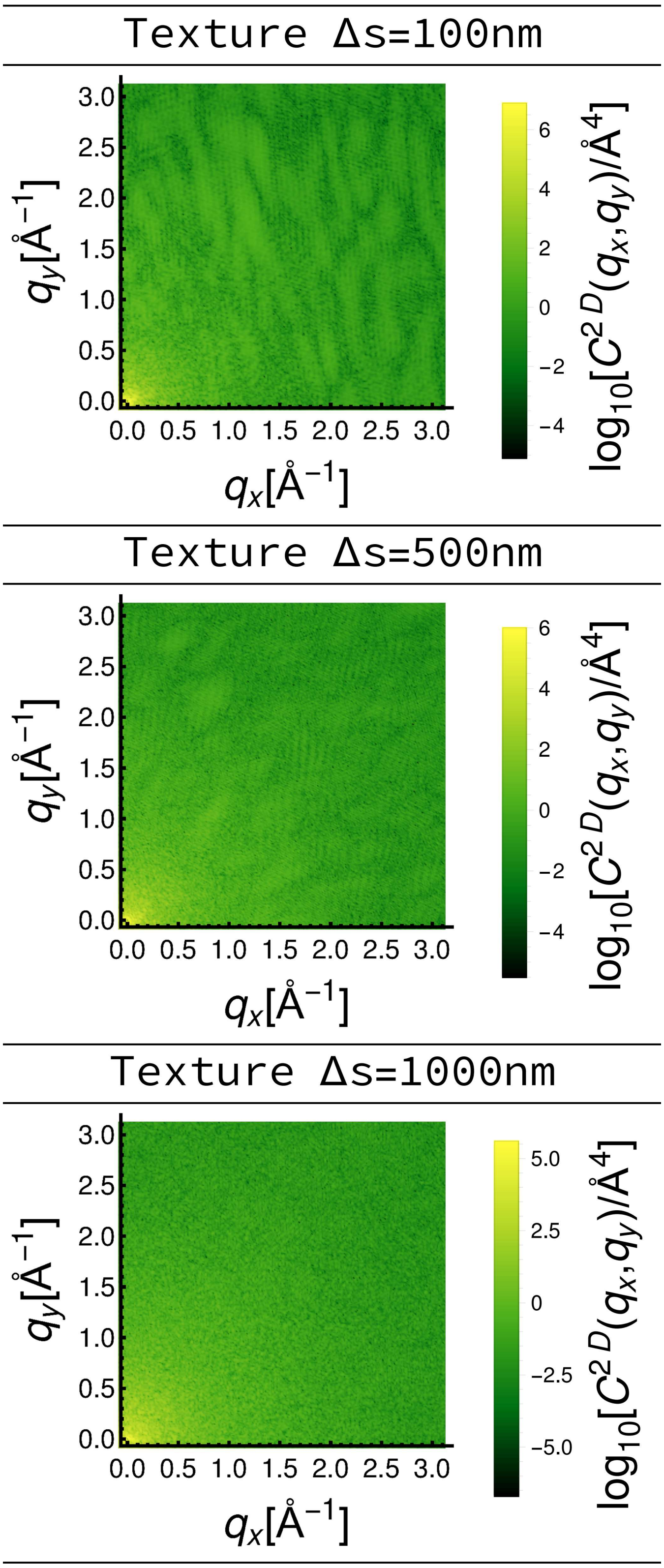

Figure C.3: Quadrant $q_x, q_x > 0$ of PSD initially-rough Si bottom surfaces (monocrystalline bulk) to assess texture evolution.

D Hurst exponent fitting

D.1 Flat surface with nanocrystalline bulk

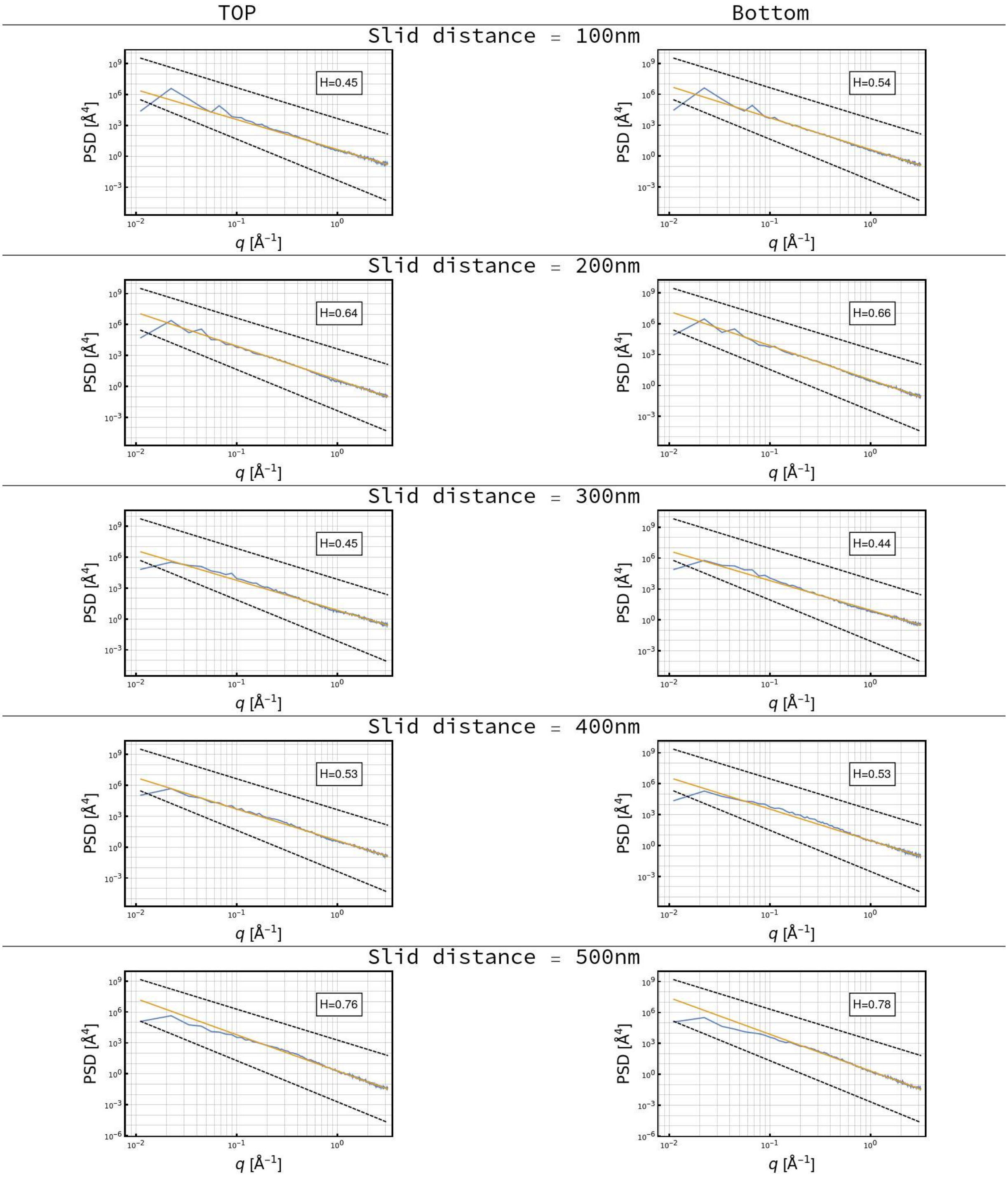

Figure D.1: Fitting the Hurst exponent in initially-flat Si-like surfaces over first sliding stages.

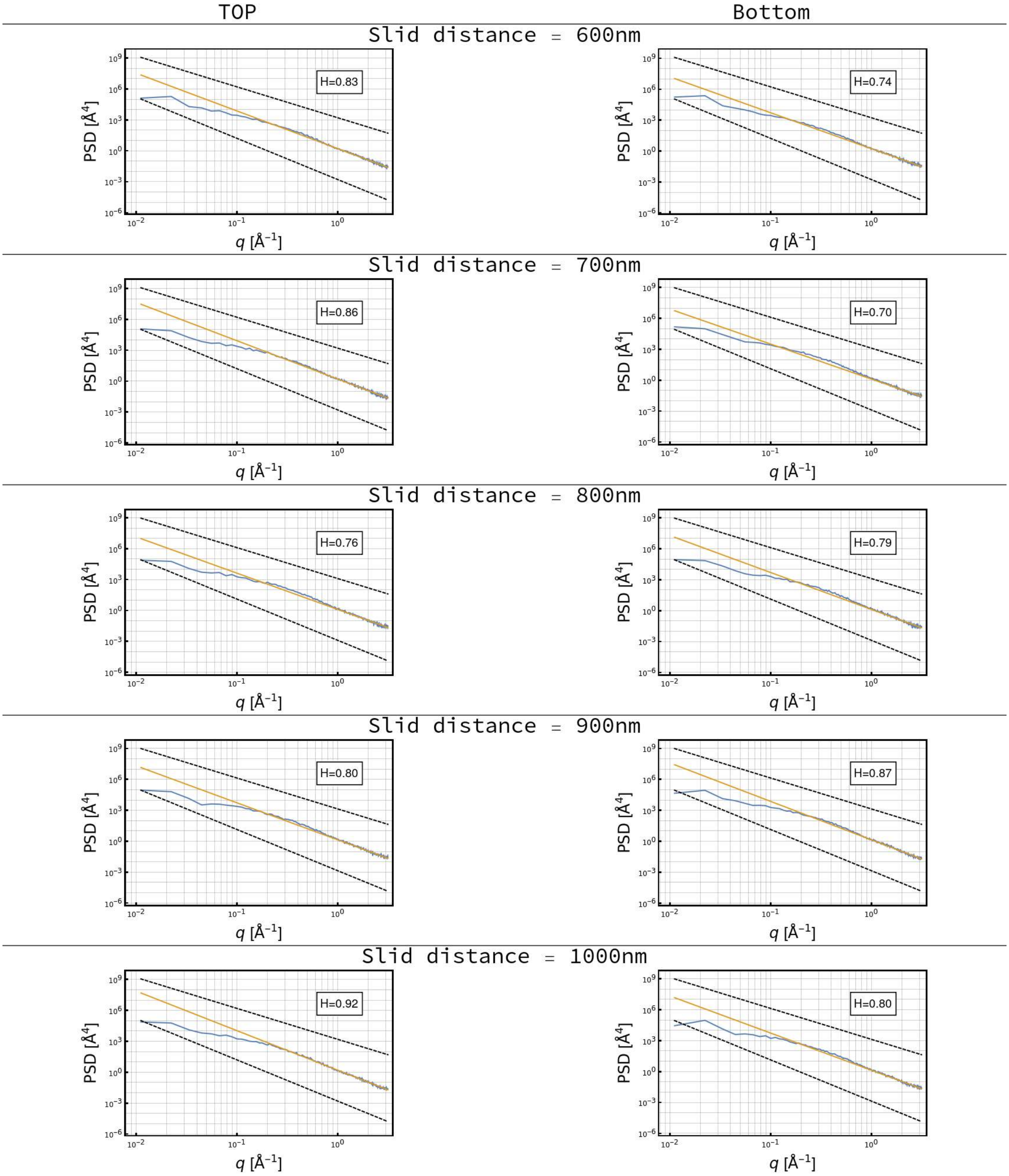

Figure D.2: Fitting the Hurst exponent in initially-flat Si-like surfaces over last sliding stages.

D.2 Rough surface with nanocrystalline bulk

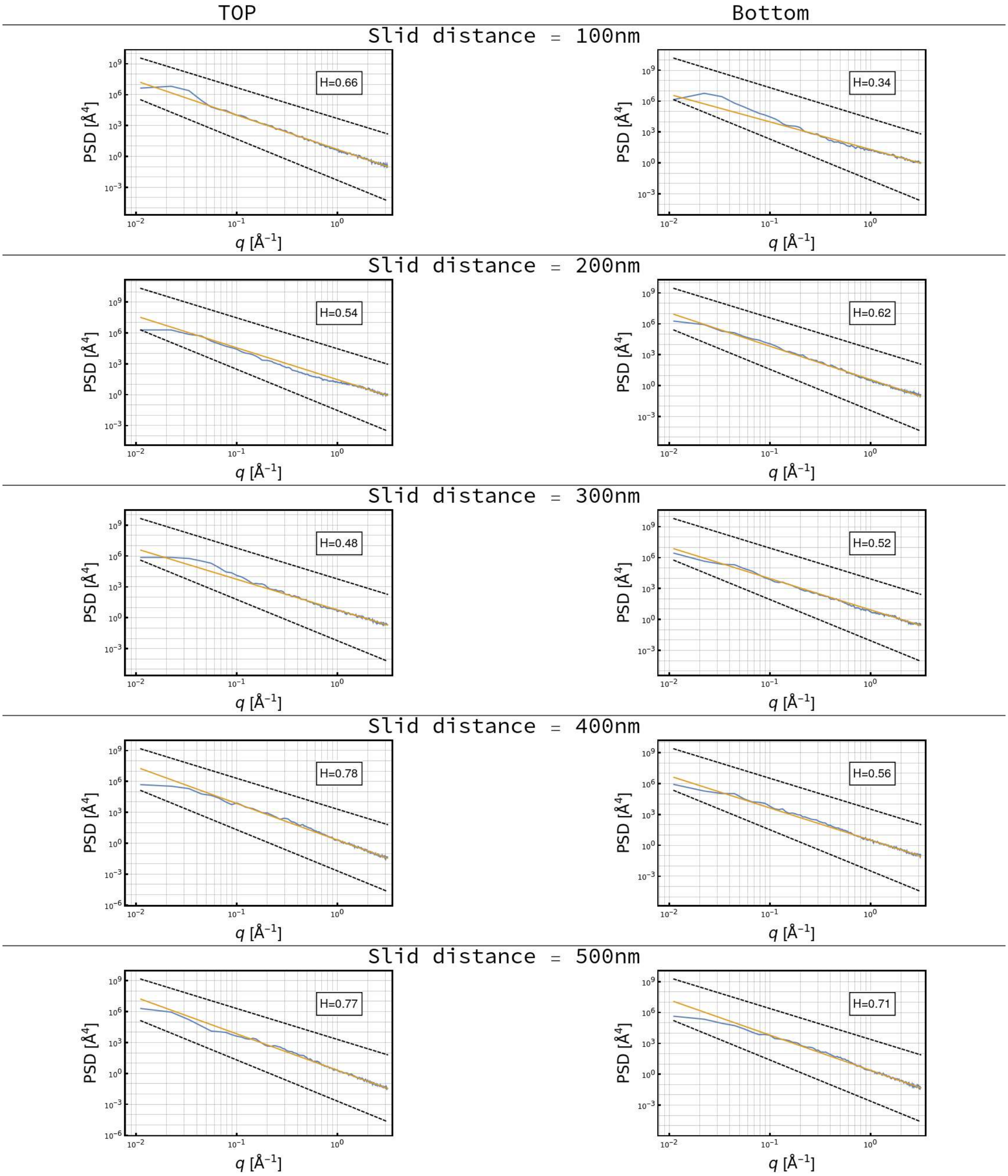

Figure D.3: Fitting the Hurst exponent in initially-rough Si-like surfaces (nanocrystalline bulk) over first sliding stages.

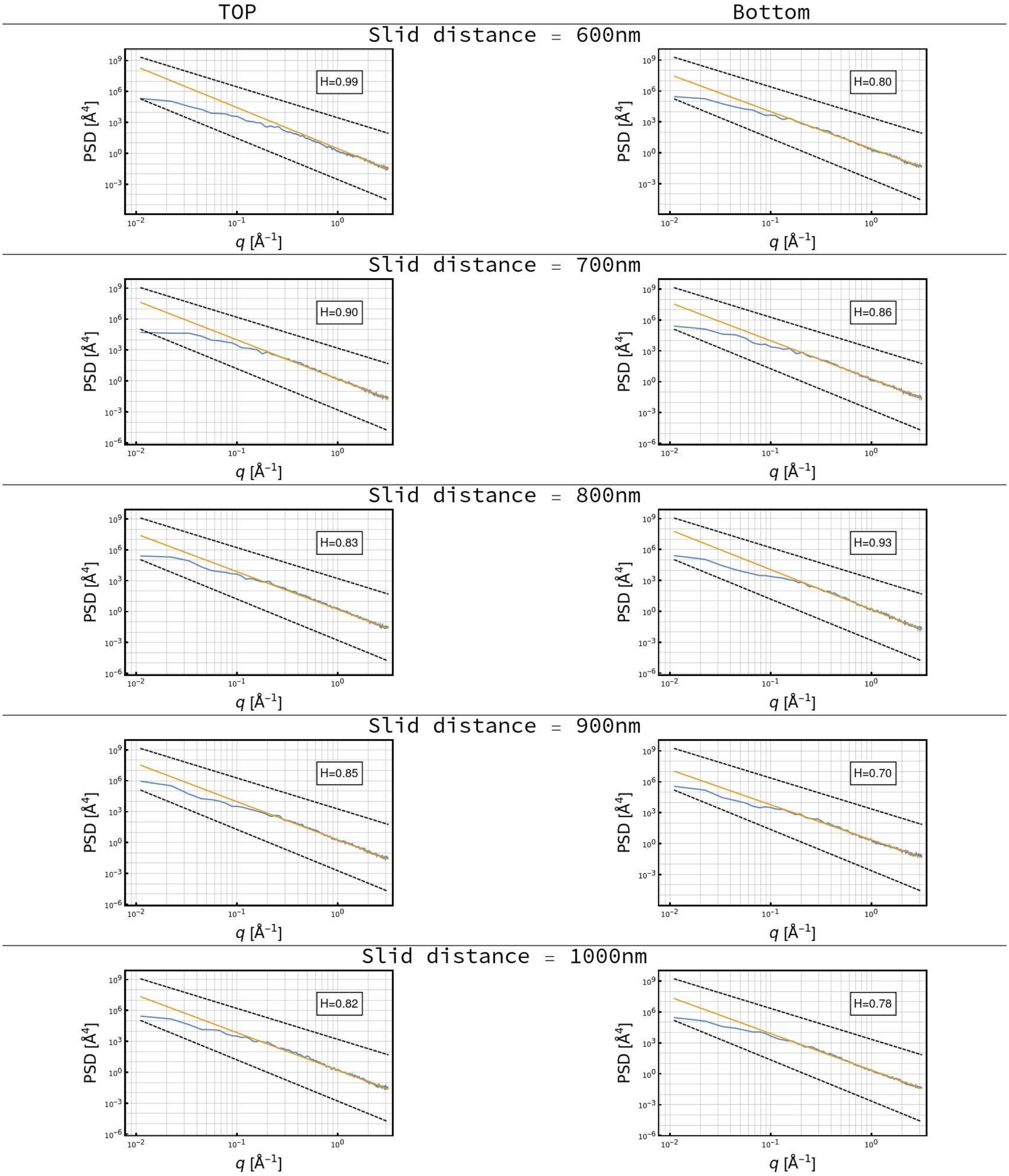

Figure D.4: Fitting the Hurst exponent in initially-rough Si-like surfaces (nanocrystalline bulk) over last sliding stages.

D.3 Rough surface with single-crystal bulk

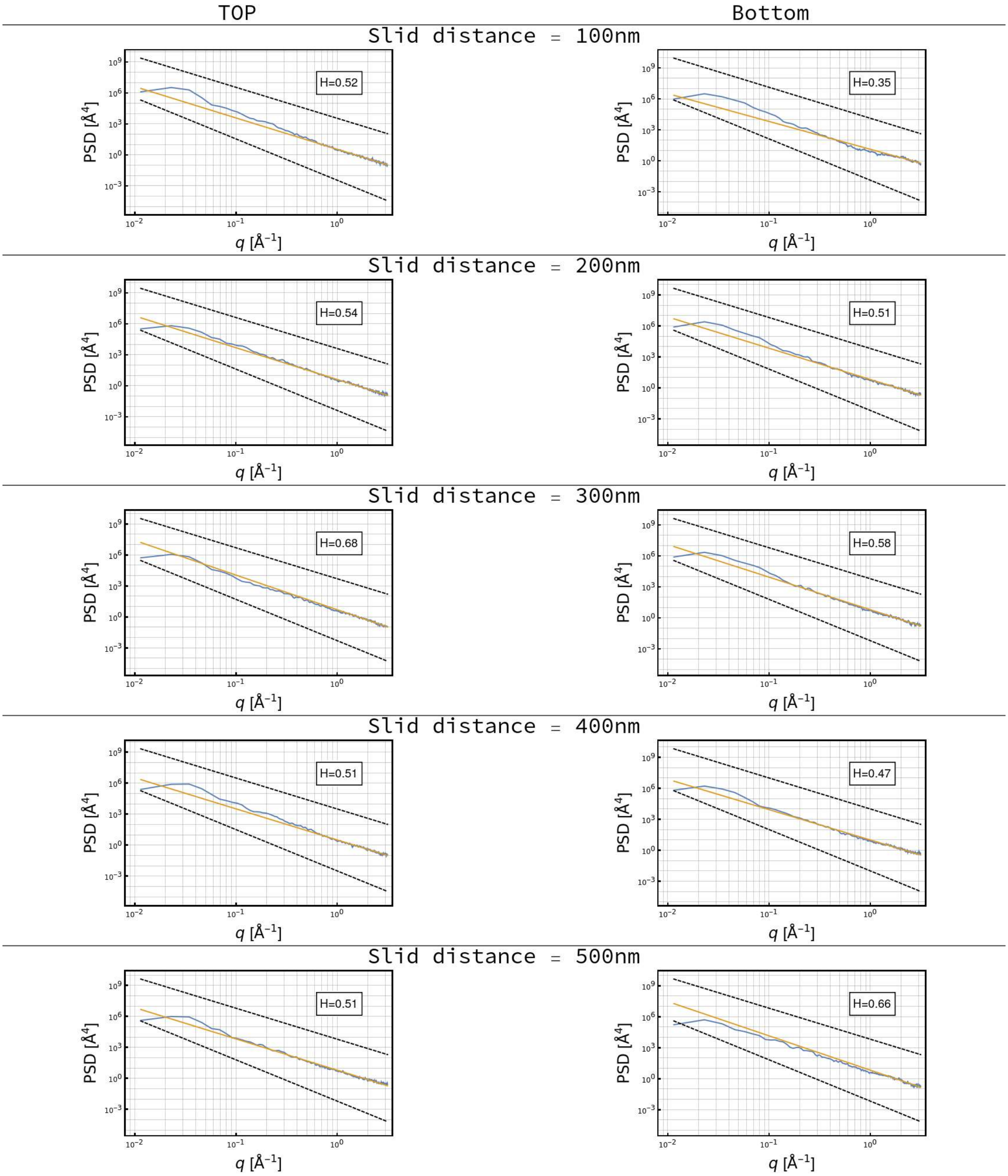

Figure D.5: Fitting the Hurst exponent in initially-rough Si-like surfaces (monocrystalline bulk) over first sliding stages.

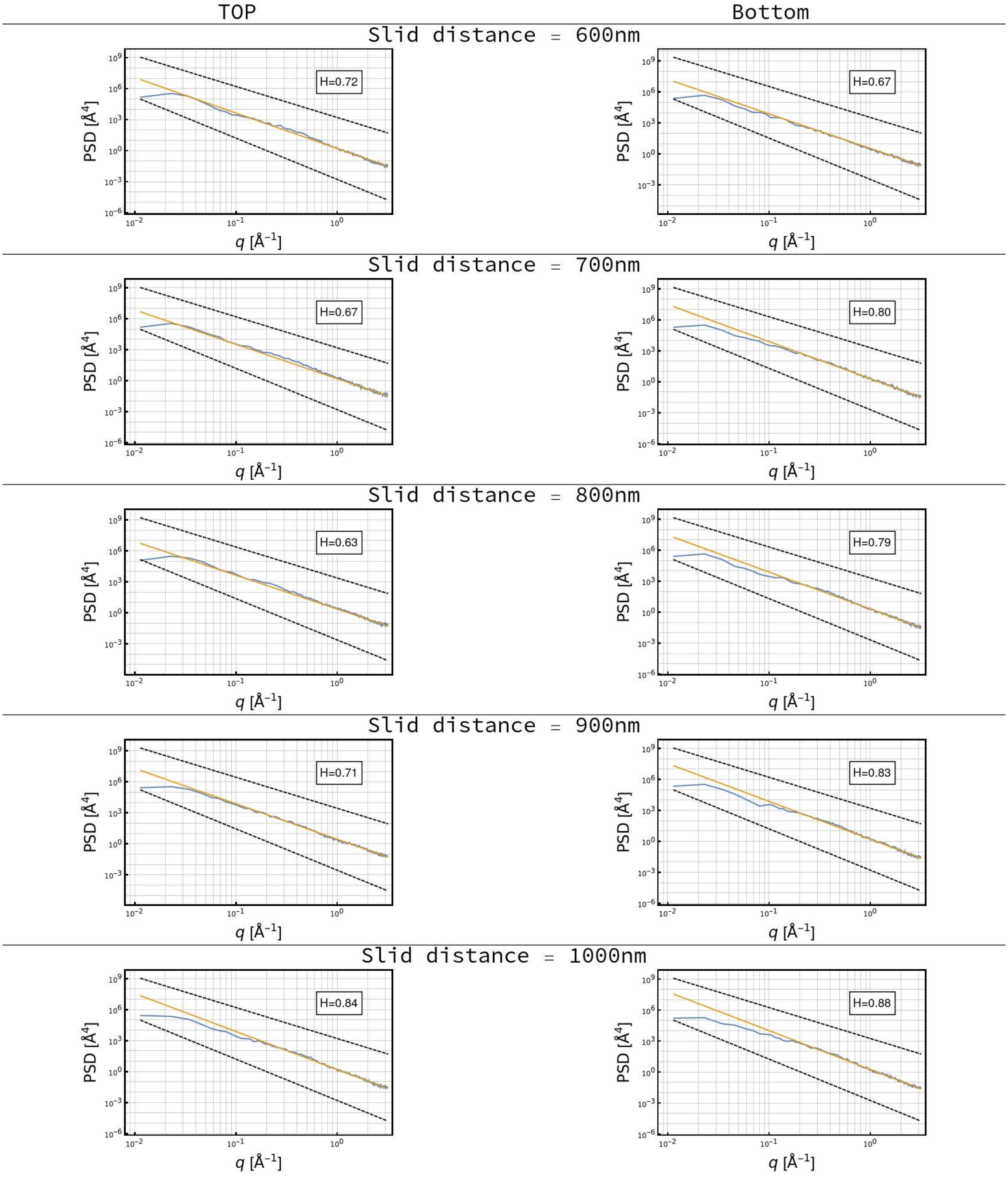

Figure D.6: Fitting the Hurst exponent in initially-rough Si-like surfaces (monocrystalline bulk) over last sliding stages.

E Simulation snapshots of flake removal

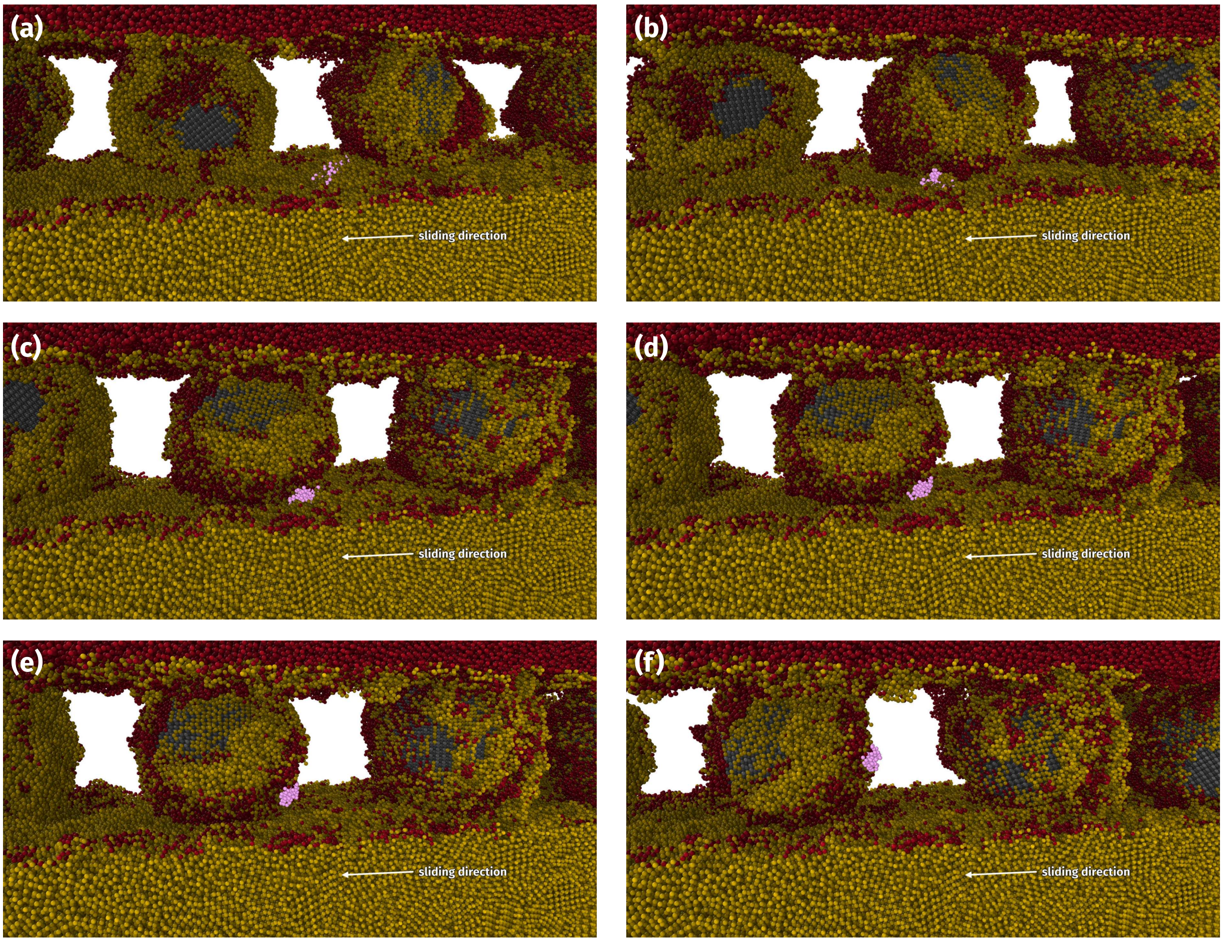

Figure E.1: The figure shows how a wear particle picks up a flake of material during the sliding process, which progresses in the panels (a)–(f). Yellow atoms originally belonged to the bottom first body, red atoms to the top one at the beginning of the simulation. The pink atoms highlight a small flake of material that detaches as a chunk during particle rolling.

F 1D scans along sliding direction and perpendicular direction

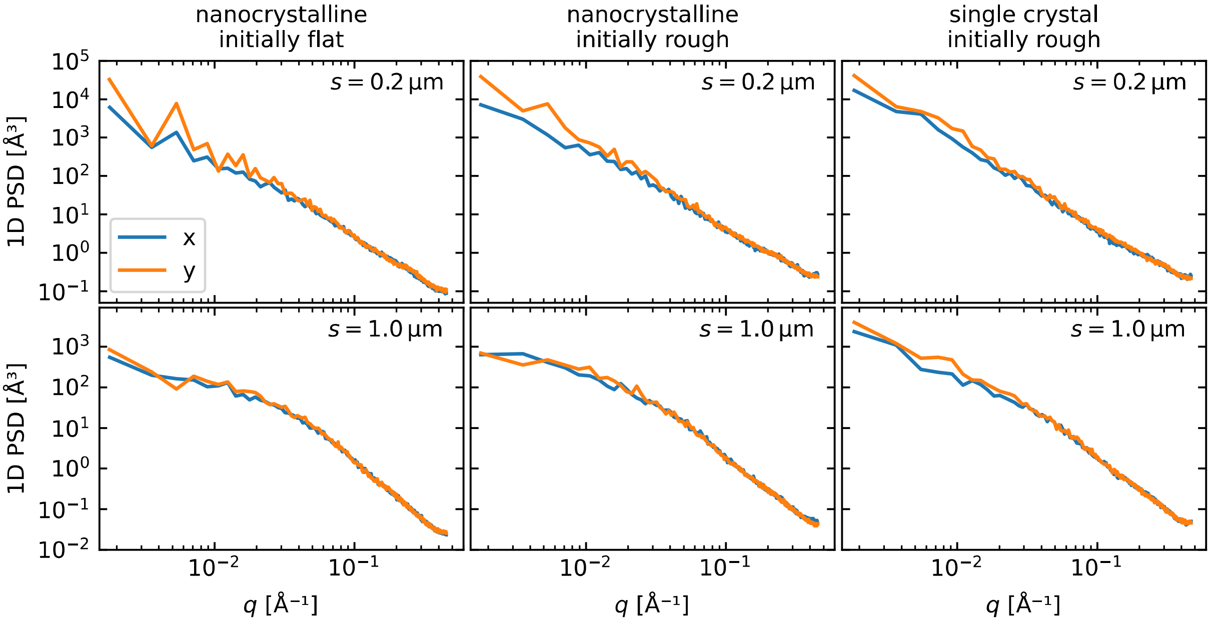

Figure F.1: 1D scans along direction of sliding ("x") and along direction perpendicular to sliding ("y"), for the three different configurations. Top row: initial state. Bottom row: final state after sliding. These results reinforce the conclusion of a final isotropic state.
